# Cryopreservation of ram epididymis spermatozoa post-slaughter - A feasible biotechnique?

**DOI:** 10.1186/1753-6561-8-S4-P166

**Published:** 2014-10-01

**Authors:** Sildivane Silva, Felipe Almeida, Ellen Silva, Helder Souza, Thalles Moura, Joane Vieira, Andreia Souza, Maria Madalena Guerra

**Affiliations:** 1 Centro de Biotecnologia (CBiotec) da Universidade Federal da Paraíba, Campus I, João Pessoa, PB, Brazil; 2 Laboratório de Andrologia (ANDROLAB) da Universidade Federal Rural de Pernambuco, Campus I, Recife, PE, Brazil; 3 Departamento de Zootecnia da Universidade Federal Rural de Pernambuco, Campus I, João Pessoa, PB, Brazil

## Background

Gametes for breeding high-value livestock is commonly preserved in gene banks for later use in assisted reproduction programs. Such material can be used in artificial insemination or embryos transfer in the field as well as embryos *in vitro *production [1]. However, when these animals die, genetic material may be lost if it is not possible to recover and preserve spermatozoa from epididymis. Thus, our aim is to establish a recovery technique of ram sperm epididymal post-slaughter and to test its post-cryopreservation viability.

## Methods

Ten rams were slaughtered and testis-epididymis of each animal was placed in individual bags and sent to the Andrology Laboratory (UFRPE). Epididymis were separated, cleaned (alcohol 70%) underwent a slicing technique and immersed in 5.0mL of Tris buffer (375mM Tris, 124mM citric acid, 41.6mM fructose, pH 7.0) for sperm migration for 10 minutes [2]. The suspension buffer-spermatozoa from each animal was evaluated for motility (0-100%) and vigor (0-5) and when approved (60%/2), diluted in Tris-yolk (20% egg-yolk, 5% glycerol, pH 7.4), packed in straws (0.25mL), cryopreserved in an automated system (TK3000^®^) and stored (-196ºC). After thawing (37ºC/30s), semen samples were evaluated for kinematic (SCA^®^) and structural integrity (plasma membrane-PI/CDF; mitochondrial membrane potential-JC1; acrosome-FITC/PNA).

## Results and conclusions

Data were expressed as mean ± sd. From 10 samples, 70% were initially approved for cryopreservation. After thawing, the following values were obtained: total motility (64.73 ± 7.10), progressive motility (19.97 ± 4.26), plasma membrane integrity (39.39 ± 4.71), mitochondrial membrane potential (88.81 ± 3.44) and acrosome integrity (49.16 ± 0.76). These results are consistent with other results of post-thaw ram semen obtained by ejaculation [3,4] as well as are indicative of function and structure preservation of sperm cell [5]. Thus, we concluded that cryopreservation of ram epididymal spermatozoa is a feasible biotechnique for germplasm conservation of animals after slaughter.
